# A Systematic Review of the Effects of Arbuscular Mycorrhizal Fungi on Root-Lesion Nematodes, *Pratylenchus* spp.

**DOI:** 10.3389/fpls.2020.00923

**Published:** 2020-07-14

**Authors:** Elaine C. Gough, Kirsty J. Owen, Rebecca S. Zwart, John P. Thompson

**Affiliations:** Centre for Crop Health, Institute for Life Sciences and the Environment, University of Southern Queensland, Toowoomba, QLD, Australia

**Keywords:** arbuscular mycorrhizal fungi, *Pratylenchus*, root-lesion nematodes, phytobiome interactions, Glomeromycota, systematic review

## Abstract

Root-lesion nematodes (*Pratylenchus* spp.) and arbuscular mycorrhizal fungi (AMF) occupy the same ecological niche in the phytobiome of many agriculturally important crops. Arbuscular mycorrhizal fungi can enhance the resistance or tolerance of a plant to *Pratylenchus* and previous studies have been undertaken to investigate the relationship between these organisms. A restructuring of the AMF phylum Glomeromycota has reallocated the species into genera according to molecular analysis. A systematic review of the literature was synthesized to assess the interaction between *Pratylenchus* spp. and AMF using the revised classification. Plants inoculated with AMF generally exhibited greater tolerance as demonstrated by increased biomass under *Pratylenchus* pressure. Species of AMF from the order Diversisporales tended to increase *Pratylenchus* population densities compared to those from the order Glomerales. Species from the genera *Funneliformis* and *Glomus* had a reductive effect on *Pratylenchus* population densities. The interaction between AMF and *Pratylenchus* spp. showed variation in responses as a result of cultivar, crop species, and AMF species. Putative mechanisms involved in these interactions are discussed.

## Introduction

*Pratylenchus* spp. or root-lesion nematodes, are migratory endoparasites (Singh et al., 2013). They feed and move through the root cortex, penetrating parenchyma cells with their stylet, excreting cell degrading enzymes, ingesting the cellular contents, and destroying cortical tissue. This results in necrotic lesions, loss of root function and consequently, reductions in plant vigor, and yield of economic products (Jones et al., 2013).

Root-lesion nematodes are polyphagous and have the broadest host range of all plant-parasitic nematodes. They are responsible for substantial yield losses of many important crop species including cereals, legumes, sugarcane, coffee, banana, potato, vegetables and fruit trees (Castillo and Vovlas, 2007). There are over 68 recognized species of *Pratylenchus* associated with the phytobiome and they are distributed in diverse habitats worldwide (Castillo and Vovlas, 2007). Historically, *Pratylenchus* spp. were distinguished on the basis of their morphometric characteristics. With the advent of molecular techniques, differences in the sequences of ribosomal DNA can distinguish between species despite high levels of intraspecific variation in some *Pratylenchus* spp. High levels of intraspecific variability occur within some *Pratylenchus* spp. such as *P. coffeae* and *P. penetrans* while other species exhibit less intraspecific internal transcribed spacer (ITS) variation, for example, *P. goodeyi* and *P. vulnus* (de Luca et al., 2011; Jones et al., 2013).

Arbuscular mycorrhizal fungi (AMF), from the phylum Glomeromycota are a ubiquitous group of soil microorganisms associated with the phytobiome. Arbuscular mycorrhizal fungi form a complex symbiosis with land plants which originated in the Ordovician period 400 million years ago (Parniske, 2008). They have remained morphologically unchanged since then, forming an intrinsic part of ecosystem functionality (Powell and Rillig, 2018). These obligate biotrophs form beneficial mutualistic associations with the roots of an estimated 80% of land plants including many agriculturally important crop species with the notable exception of most species in the families *Brassicaceae* and *Chenopodiaceae* (Lambers and Teste, 2013). Their characteristic arbuscules (microscopic tree-like structures) within the root cortical cells of compatible plants enable the photosynthetically derived organic compounds supplied by the plant to be exchanged for inorganic nutrients and water supplied by the fungus from the soil. The fungus also aids in the stabilization of soil aggregates through hyphal binding and exudation of glomalin (Smith and Read, 2008; Leifheit et al., 2014). It is estimated that up to 20% of the photosynthetic carbon of plants is allocated to maintaining the fungal association (Smith and Read, 2007). This carbon cost to the plant is outweighed by the many benefits conferred by the fungi, foremost of which are improved acquisition by the fungal hyphae of immobile nutrients from the soil such as phosphorus (P) and zinc (Zn) (Parniske, 2008).

Arbuscular mycorrhizal fungi have been promoted as a natural tool to maintain and promote sustainable agriculture due to their role as natural biofertilizers; increasing the levels of nitrogen (N), P and Zn in the crop (Thompson, 1993; Parniske, 2008; Smith et al., 2011; Baum et al., 2015; Berruti et al., 2016). They also play a role in drought tolerance (Zhao et al., 2015) and as bio-protectants against fungal, bacterial, and nematode pathogens (Whipps, 2004; Pozo and Azcón-Aguilar, 2007; Veresoglou and Rillig, 2012; Yang et al., 2014).

Early classifications defined species within the order Glomerales of the phylum Glomeromycota on the basis of spore morphology (Morton and Benny, 1990). Schüßler and Walker (2010) restructured the phylum Glomeromycota according to molecular phylogenies based on the small subunit (SSU) rRNA gene, the large subunit (LSU) rRNA gene, β-tubulin sequence data and the ITS region. Consequently, the current classification of the order Glomerales consists of two families — the *Glomeraceae* and the *Claroidoglomeraceae*. A number of *Glomus* species have been transferred to the genera *Funneliformis* and *Rhizophagus*. Table 1 shows the phylum Glomeromycota and the subdivisions into the orders Glomerales, Diversisporales, Archaeosporales, and Paraglomerales (Redecker et al., 2013).

**Table 1:** Classification of the phylum Glomeromycota according to Redecker et al. (2013).

**Order**	**Family**	Genus^*^
Diversisporales	Diversisporaceae	*Tricispora*
		*Otospora*
		*Diversispora*
		*Corymbiglomus*
		*Redeckera*
	Acaulosporaceae	***Acaulospora***
	Sacculosporaceae	*Sacculospora*
	Pacisporaceae	*Pacispora*
	Gigasporaceae	***Scutellospora***
		***Gigaspora***
		*Intraornatospora*
		*Paradentiscutata*
		***Dentiscutata***
		*Centraspora*
		*Racocetra*
Glomerales	Claroideoglomeraceae	***Claroideoglomus***
	Glomeraceae	***Glomus***
		***Funneliformis***
		*Septoglomus*
		***Rhizophagus***
		*Sclerocystis*
Archaeosporales	Ambisporaceae	*Ambispora*
	Geosiphonaceae	*Geosiphon*
	Archaeosporaceae	*Archaeospora*
Paraglomerales	Paraglomeraceae	*Paraglomus*

* *Genera in bold were considered in this review*.

Plant-parasitic nematodes are classified according to their feeding strategies. These include (i) ecto-parasitic nematodes which feed externally on root cells and remain in the rhizosphere such as *Tylenchorhynchus* spp., (ii) migratory endo-parasitic nematodes which enter the plant root, feed, and move through the root tissues destroying cells as they migrate such as *Pratylenchus* spp., and, (iii) sedentary endo-parasitic nematodes which convert vascular cells into specialized feeding cells where they remain, such as the root-knot nematodes (*Meloidogyne* spp.) and the cyst nematodes (*Heterodera* and *Globodera* spp.) (Decraemer and Hunt, 2013).

The coexistence of AMF and nematodes in the phytobiome has prompted a number of investigations into their interactive effects on plants (reviews: Pinochet et al., 1996; meta-analyses: Borowicz, 2001; Hol and Cook, 2005; Veresoglou and Rillig, 2012; Yang et al., 2014). Published meta-analyses describe the generally suppressive effect that AMF have on nematodes (Veresoglou and Rillig, 2012; Yang et al., 2014). These analyses included nematodes belonging to different genera and they grouped plant-parasitic nematodes into their feeding modes (sedentary or migratory). AMF reduced the numbers of the sedentary endo-parasitic nematodes (*Meloidogyne, Heterodera*, and *Globodera* spp.) and the ectoparasitic nematodes (*Tylenchorhynchus* spp.). However, some analyses showed an increase in migratory endo-parasitic nematode numbers on inoculation with AMF (Borowicz, 2001; Hol and Cook, 2005). Grouping the nematodes into their broad feeding modes has the effect of obscuring the data on interactions of AMF with *Pratylenchus* spp. and those with other migratory endo-parasites including *Radopholus* spp. and *Hirschmanniella* spp.

Due to the ubiquitous distribution and the great economic importance of *Pratylenchus* spp. to agricultural crops worldwide, this systematic review examines the relationship exclusively between *Pratylenchus* spp. and AMF taking into account the current classification of AMF genera. All life stages of *Pratylenchus* spp., adults, juveniles, and eggs occupy the same root cortex tissue as the AMF structures of hyphae, arbuscules, and vesicles (Pinochet et al., 1996) and co-occur with AMF extraradical hyphae and spores in the rhizosphere soil.

The aims of this review are to determine (a) the responses in *Pratylenchus* population densities to AMF, (b) the effects of AMF on the growth of plants infested with *Pratylenchus* and, (c) the effects of degree of AMF colonization on *Pratylenchus* population density. The outcomes of the systematic review are discussed in relation to putative mechanisms involved in the interaction between *Pratylenchus* spp. and AMF. These mechanisms may include: (a) enhanced plant tolerance to *Pratylenchus* as a result of increased nutrient uptake and altered root morphology, (b) direct competition between *Pratylenchus* and AMF for resources and space, (c) effects on *Pratylenchus* through plant defense mechanisms such as induced systemic resistance in the plant from AMF colonization, and (d) altered rhizosphere interactions (Pozo and Azcón-Aguilar, 2007; Schouteden et al., 2015).

## Methods

### Selection of Studies

A systematic review of the literature was performed according to PRISMA systematic review guidelines (Moher et al., 2009). Studies investigating interactions between *Pratylenchus* spp. and AMF were obtained from the databases,—Web of Science (www.webofknowledge.com), SCOPUS (https://www.scopus.com) and Google Scholar (https://scholar.google.com/).

The search parameters included the following terms, “*Pratylenchus*,” “arbuscular mycorrhizal fungi” AND “root-lesion nematode.” The papers were further screened to select original research with quantitatively measured data of the following response variables: (a) effects of AMF on *Pratylenchus* population densities, (b) effects of *Pratylenchus* spp. on degree of AMF colonization in the roots (mycorrhization), and (c) effects of both organisms on plant biomass. Other pre-requisites for eligibility for inclusion in the review were (a) studies with one or more AMF species, but not mixed treatments with other beneficial organisms, (b) studies with *Pratylenchus* species alone not mixed with other plant-parasitic nematodes, and (c) studies with a non-inoculated control. Reviews, meta-analyses and book chapters were excluded from the analyses, but the original research papers cited within were cross referenced and assessed for suitability for inclusion.

### Analyses of Response Variables

The “nematode response” was calculated using the following formula:

(1) $$\begin{array}{l} nematode\;response=\frac{\left(Pratylenchus-Pratylenchus\;plus\;AMF\right)}{Pratylenchus}*100 \end{array}$$

where “*Pratylenchus*” is the final population density of *Pratylenchus* in nematode only treatments and “*Pratylenchus plus AMF*” is the population density of *Pratylenchus* in co-inoculated AMF and nematode treatments.

The “biomass response” was calculated using the following formula:

(2) $$\begin{array}{l} biomass\;response\\ \;=\;\frac{\left(Pratylenchus\;biomass-Pratylenchus\;plus\;AMF\;biomass\right)}{Pratylenchus\;biomass}*100 \end{array}$$

Where “*Pratylenchus biomass*” is the plant biomass in nematode only inoculated treatments and “*Pratylenchus plus AMF biomass*” is the plant biomass in co-inoculated AMF and nematode treatments. Biomass data were expressed as shoot, root and total biomass where available.

The “AMF response” was calculated using the following formula:

(3) $$\begin{array}{l} AMF\;response=AMF\;\%\;colonisation-AMF\;\%\;colonisation\;plus\;Pratylenchus \end{array}$$

where “*AMF % colonization*” is the percentage of mycorrhization of plants with AMF alone and “*AMF % colonization plus Pratylenchus*” is the percentage of mycorrhization of plants co-inoculated with AMF and nematodes.

The effect of inoculation with AMF on the *Pratylenchus* population density was categorized as decrease, no effect, or increase based on statistical significance (*P*<0.05) of studies in the original publications. A chi-squared test for independence was performed to assess the relationship between order of AMF (Glomerales and Diversisporales) and effect on *Pratylenchus* population densities. Chi-squared values were calculated from two-way contingency tables (Steel and Torrie, 1960) of AMF order by *Pratylenchus* density effect for the 56 studies using the following function:

(4) $$\begin{array}{l} \chi^{2}=\Sigma \left\{{\left(\text{observed number}-\text{expected number}\right)}^{2}/\text{expected number}\right\} \end{array}$$

The percentage AMF colonization of the roots of the plants in these three categories of AMF effects on *Pratylenchus* population densities for the studies with relevant data was subjected to one-way analysis of variance (ANOVA) using GenStat (VSN International, 2014).

The data were examined under other independent groupings such as (a) restructured AMF genera according to the current classification by Schüßuler and Walker (Schüßler and Walker, 2010) and (b) host plant functional group (grasses, trees, herbs, shrubs).

## Results

The initial search conducted on all available literature in the three databases provided 519 potential papers for inclusion. Further screening by removing duplicates and ineligible papers resulted in 22 full text articles selected for the systematic review (Table 2). Experiments within papers were treated as separate studies when; (a) two or more AMF species were studied independently, (b) more than one plant cultivar was included, and (c) more than one time of inoculation was used. If there were various times of assessment for plant biomass over multiple years, the most recent data set was used. In total, 60 studies were analyzed (Supplementary Table 1).

**Table 2:** PRISMA Flow Diagram for eligible articles to include in the qualitative review.

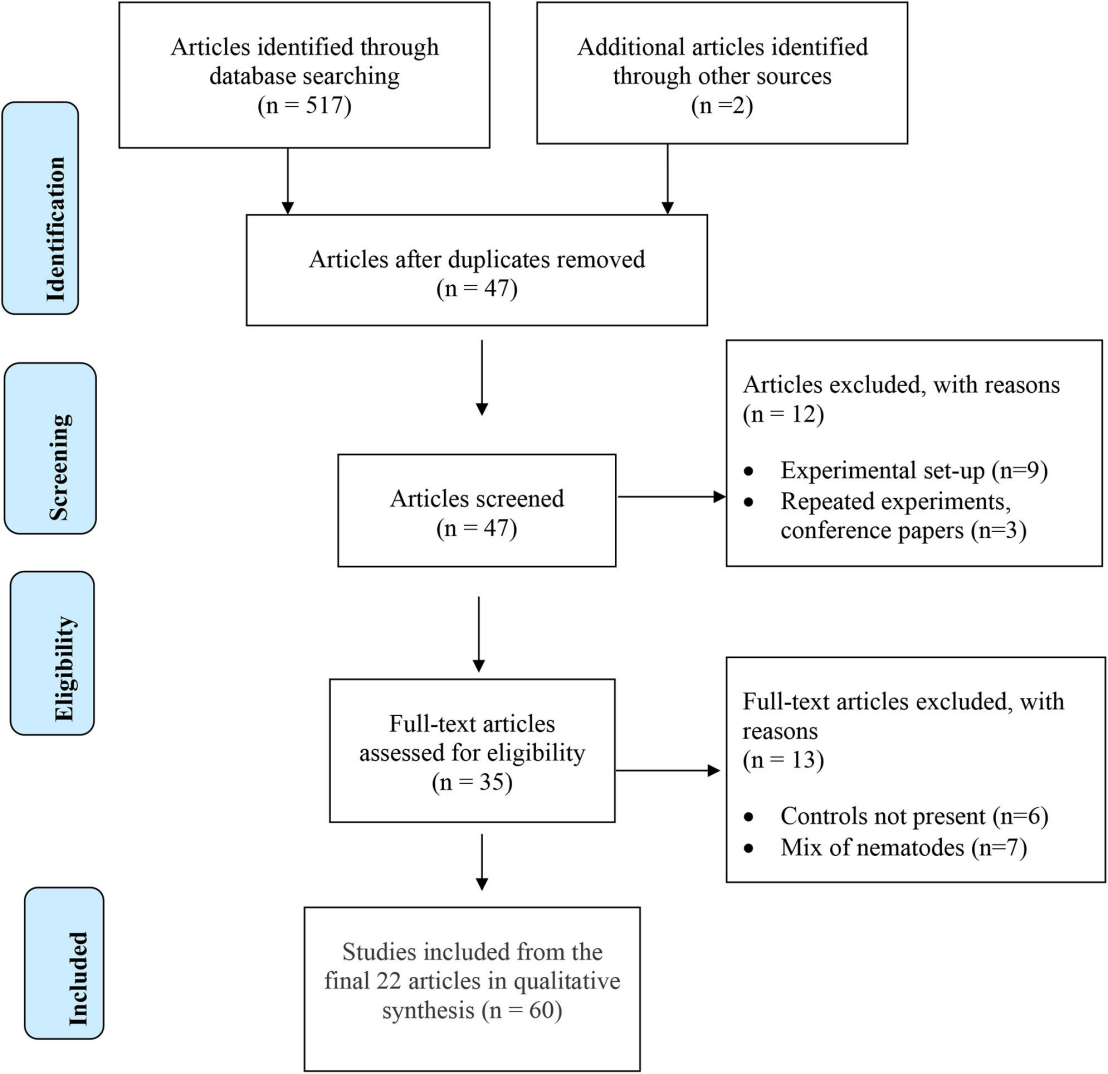

Table 3 shows the response of *Pratylenchus* sp., arbuscular mycorrhizal fungi (AMF) and plants to co-inoculation of AMF and *Pratylenchus* sp. compared to *Pratylenchus* sp. alone in glasshouse and microplot experiments. The data is statistically significant as stated in the original papers. The majority of the crops assessed were agriculturally or horticulturally important with the exception of dune grass (*Ammophilia arenaria*). In general, the experiments were undertaken in glasshouses with some transplanting of pre-inoculated AMF colonized plants to field microplots. There were 14 individual species of AMF used in 43 studies, one undetermined species in ten studies, and a mix of AMF species in seven studies. These species came from both the order Glomerales which included the genera *Rhizophagus, Glomus, Funneliformis, Claroideoglomus*, and the order Diversisporales, which included the genera *Acaulospora, Dentiscutata, Gigaspora*, and *Scutellospora*.

**Table 3:** Response of *Pratylenchus* spp., arbuscular mycorrhizal fungi (AMF) and plants to co-inoculation of AMF and *Pratylenchus* spp. compared to *Pratylenchus sp*. alone in glasshouse and microplot experiments.

**Plant species (common name)**		***Pratylenchus* species**	**Response to AMF-*****Pratylenchus*** **interaction (%)**					
	**AMF species**		**Nematode**	**AMF**	**Biomass**	**Shoot wt**	**Root wt**	**Reference**
**GRASS**								
*Triticum aestivum* (wheat)	Mix: *Claroideoglomus etunicatum, F. coronatum, Rhizophagus irregularis, F. mosseae*	*P. neglectus*	47 to 117^1^	ns	↓30 to ↓40^1^	ND	↓31 to ↓44^1^	Frew et al., 2018
*Zea mays* (maize)	*R. clarus*	*P. brachyurus*	990	ND	ND	ns	ND	Brito et al., 2018
	*Dentiscutata heterogama*		353	ND	ND	ns	ND	
	*Gigaspora rosea*		447	ND	ND	ns	ND	
	*C. etunicatum*		441	ND	ND	ns	ND	
	*G. margarita*		353	ND	ND	ns	ND	
	*S. calospora*		900	ND	ND	ns	ND	
*Ammophila arenaria* (dune grass)	*Glomus* sp.	*P. dunensis*	↓38^2^	ns	↓44^2^	ND	ND	Rodríguez-Echeverría et al., 2009
	*Glomus* sp.	*P. penetrans*	↓67^2^	ns	ns	ND	ND	
	Mix: *Glomus* spp., *S. castanea*	*P. penetrans*	↓47 to ↓86^3^	ns	ns	ND	ns	de La Peña et al., 2006
Tree								
*Cydonia oblonga* (quince)	*R. intraradices*	*P. vulnus*	ns	↓26	ND	65	51	Calvet et al., 1995
*Malus domestica* (apple)	*C. claroideum*	*P. penetrans*	ns	ND	ND	ND	ns	Ceustermans et al., 2018
	*Acaulospora longula*		ns	ND	ND	ND	ns	
	*C. claroideum, A. longula*		ns	ND	ND	ND	165	
	*R. intraradices*		ns	ND	ND	ND	ns	
	AMF species mix (13)		↓97	ND	ND	ND	ns	
	*C. etunicatum*		ns	ns	ns	8	ns	Forge et al., 2001^^*^^
	*R. aggregatus*		ns	ns	ns	ns	ns	
	*R. clarus*		ns	ns	ns	ns	ns	
	*F. mosseae*		ns	ns	19 to 45^1^	9 to 54^1^	1 to 32^1^	
	*R. intraradices*		ns	ns	19 to 43^1^	12 to 49^1^	5 to 37^1^	
	*G. versiforme*		ns	ns	ns	47	ns	
*Malus silvestris* (crab apple)	*F. mosseae*	*P. vulnus*	↓51	ns	ND	201	142	Pinochet et al., 1993
*Pyrus communis* (pear)	*R. intraradices*		↓57	ns	ND	403	209	Lopez et al., 1997
	*F. mosseae*		↓63	ns	ND	341	202	
*Prunus mahaleb* (cherry)	*R. intraradices*		ns	ns	ND	89	78	Pinochet et al., 1995a
*Prunus persica* (peach)	*F. mosseae*		↓42	ns	ND	ns	ns	Pinochet et al., 1995b
*Prunus cerasifera* X *P. munsoniana* (Prunus rootstock)	*R. intraradices*		ns	↓14	ND	ns	28	Pinochet et al., 1998
	*F. mosseae*		ns	↓14	ND	ns	ns	
*Prunus cerasifera* (cherry plum)	*F. mosseae*		ns	↓34^1^	ND	ns	86^1^	Camprubi et al., 1993
Herb								
*Musa* sp. (banana)	*F. mosseae*	*P. coffeae*	↓76^1^	ns	ND	175 to 433^1^	192 to 310^1^	Elsen et al., 2003a
	*F. mosseae*		↓79 to ↓80^1^	↓17 to ↓24^1^	ND	ns	ns	Elsen et al., 2003b
	*F. mosseae*	*P. goodeyi*	ns	ND	ND	16	ns	Jaizme-Vega and Pinochet, 1997
	*R. aggregatus*		ns	ND	ND	14	ns	
	*R. intraradices*		ns	ND	ND	8	ns	
*Phaseolus vulgaris* (common bean)	*R. fasciculatus*	*P. penetrans*	ns	ND	ND	ND	ND	Elliott et al., 1984
*Daucus carota* (carrot)	*F. mosseae*	*P. penetrans*	↓48	ns	207	ND	ND	Talavera et al., 2001
*Lycopersicon esculentum* (tomato)	*F. mosseae*		↓87	ns	ND	ns	ns	Vos et al., 2012
*Ananas comosus* (pineapple)	*Glomus* sp.	*P. brachyurus*	↓24 to ↓74^4^	↓9 to ↓32^4^	ND	105 to 359^4^	50 to 269^4^	Guillemin et al., 1994
Shrub								
*Gossypium hirsutum* (cotton)	*Gigaspora margarita*	*P. brachyurus*	↓66	ND	ND	556	544	Hussey and Roncadori, 1978
*Coffea arabica* (coffee)	*A. mellea*		1049^3^	↓32^3^	946^3^	ND	ND	Vaast et al., 1997
	*R. clarus*		432^3^	↓26^3^	504^3^	ND	ND	

1 Cultivar dependent;

2 AMF, country of origin dependant;

3 Time of inoculation dependent;

4 Cultivar and time of inoculation dependent; ns, non-significant result; ND, not determined. Nematode response, difference between Pratylenchus alone and co-inoculated with AMF; AMF response, difference between percentage mycorrhization of AMF alone and co-inoculated with Pratylenchus; Biomass response; difference between Pratylenchus alone and co-inoculated with AMF; ↓ indicates negative effect of AMF x Pratylenchus interaction;

* *Glasshouse data only*.

The studies involved seven *Pratylenchus* spp. namely *P. penetrans, P. vulnus, P. neglectus, P. coffeae, P. goodeyi, P. brachyurus* and *P. dunensis*. These species reviewed are many of the species of *Pratylenchus* causing the most economic damage worldwide (Jones and Fosu-Nyarko, 2014).

### Responses in *Pratylenchus* Population Densities to AMF

The effects of AMF inoculation on *Pratylenchus* population densities varied from a decrease in population densities (*n* = 22), no effect on *Pratylenchus* population densities (*n* = 28), to an increase in *Pratylenchus* population densities (*n* = 10).

The taxonomic order of AMF species used had an effect on *Pratylenchus* densities, whereby inoculation with species from the order Glomerales tended to decrease *Pratylenchus* population densities compared with species from the order Diversisporales which tended to increase *Pratylenchus* population densities (Table 4). Although there were fewer studies with comparisons for Diversisporales than for Glomerales, the differences in response between these groupings were highly significant (Table 4). Within the Glomerales, inoculation with the genera *Glomus* and *Funnelifomis* had a neutral to reductive effect on *Pratylenchus* population densities.

**Table 4:** Number of studies investigating AMF-*Pratylenchus* interaction included in the systematic review and the effect of AMF order on *Pratylenchus* populations.

**Order**	**Genus**	**Effect on** ***Pratylenchus*** **populations**			**Total studies**
		**Increase**	**No effect**	**Decrease**	
Glomerales	*Rhizophagus*	2	12	1	15
	*Glomus*	0	2	8	10
	*Funneliformis*	0	8	8	16
	*Claroideoglomus*	1	2	0	3
	AMF mix (*Claroideoglomus, Rhizophagus, Funneliformis*)	2	0	1	3
	Total	5	24	18	47
Diversisporales	*Acaulospora*	1	2	0	3
	*Dentiscutata*	0	1	0	1
	*Gigaspora*	2	0	1	3
	*Scutellospora*	2	0	0	2
	Total	5	2	2	9
		χ2 = 10.43 with 2 d.f. P < 0.01			

Increases in *Pratylenchus* population densities due to AMF inoculation in studies subdivided in relation to the host plant functional group were predominantly found in the grasses (increases in 8 out of 15 studies). No increase in *Pratylenchus* population densities were found in trees (0 increases in 24 studies), or herbs (0 increases in 16 studies).

### Effects of AMF on the Growth of Plants Infested With *Pratylenchus*

Plant shoot biomass increased when AMF were co-inoculated with *Pratylenchus* compared with infection with *Pratylenchus* alone. From the 34 studies with data providing comparisons on shoot biomass, 24 showed an increase in shoot biomass while 10 had no effect. No studies showed a reduction in shoot biomass. Most studies calculated shoot biomass (*n* = 35) and root biomass independently (*n* = 41), with fewer reporting results on total biomass (*n* = 28). From these 28 studies, eight showed an increase in total plant biomass, and three studies a decrease in total plant biomass with 17 having no significant effect.

The change in root biomass between plants inoculated with *Pratylenchus* and the plants co-inoculated with AMF and *Pratylenchus* is shown in Table 3. The majority of the studies showed an increase in root biomass when inoculated with AMF (*n* = 22) in the presence of *Pratylenchus* with the exception of two studies by Frew et al. (2018).

### Effects of Degree of AMF Colonization on *Pratylenchus* Population Density

There were 58 studies with data on the degree of AMF colonization of the roots. In most studies there was a decrease (*n* = 21) or no effect (*n* = 27) on *Pratylenchus* population densities, which were associated with relatively high percentage AMF colonization of the roots (43.9 and 42.2% respectively), compared to an increase in *Pratylenchus* population densities (*n* = 10), which were associated with a significantly lower percentage AMF colonization (20.1%) (Table 5).

**Table 5:** Effects of AMF inoculation on change in *Pratylenchus* population densities in relation to degree of AMF colonization in the roots.

**Change in *Pratylenchus* population density**	**Number of comparisons**	**AMF % colonization in presence of** ***Pratylenchus***		
		**log_e_**	**SE^a^**	**BTM (%)^b^**
Decrease	21	3.7818	0.1419	43.9
No effect	27	3.7421	1.1252	42.2
Increase	10	2.9994	0.2057	20.1

*Fprobability, 0.006 from ANOVA of the transformed data*.

a *SE, standard error*.

b *BTM, back-transformed mean*.

## Discussion

This review is the first to examine the effects of specific genera and order of AMF acting on *Pratylenchus* population densities and demonstrates that the taxonomic order of AMF has a significant influence on *Pratylenchus* population densities. Previous reviews and meta-analyses showed a varied response of AMF on migratory endo-parasites ranging from a suppressive (Veresoglou and Rillig, 2012; Yang et al., 2014) to a stimulatory effect (Borowicz, 2001; Hol and Cook, 2005).

Variation in functionalities between AMF families has been reported (Smith et al., 2004). Members of the Glomeraceae are typically fast colonizers, concentrating their hyphae within the plant roots and can increase P uptake and promote plant growth under pathogen attack and drought stress (Klironomos, 2000; Hart and Reader, 2002; Maherali and Klironomos, 2007; Yang et al., 2015; Seymour et al., 2019). Members of the Diversisporales are typically slower to colonize roots, concentrating hyphae externally to the plant root in the soil and are effective at enhancing plant phosphorus uptake (Klironomos, 2000; Hart and Reader, 2002; Maherali and Klironomos, 2007). However, from the studies in this review, there was lack of data on the percentage of AMF colonization of the controls in the order Diversisporales (*n* = 2) therefore it remains unclear if Diversisporales are slower to colonize from these studies.

From our review, species from the genera *Glomus* or *Funneliformis*, in the order Glomerales decreased or had no significant effect on the *Pratylenchus* population densities compared with *Rhizophagus* and *Claroideoglomus*. The difference in effects that AMF genera have on *Pratylenchus* population densities could be due to differences in the secondary metabolites produced under the symbiotic relationship. For example, in tomato, although the metabolic pathways altered by the AMF symbiosis were similar, different metabolites were produced, depending on inoculation with *F. mosseae* or *R. irregularis* (Pozo et al., 2002). An increase in the accumulation of bioactive forms of jasmonic acid was found in roots colonized by *F. mosseae* (Rivero et al., 2015). Jasmonic acid and its derivative methyl jasmonate play a role in plant defense against herbivores and they can reduce susceptibility of plants to infestation by *Pratylenchus* (Soriano et al., 2004). Root metabolites may influence populations of plant parasitic nematodes by acting as attractants, repellents or affecting hatch rates of nematodes (Sidker and Vestergård, 2019). Mycorrhizal colonization can increase phenolics such as ferulic acid and gallic acid in the host plants (López-Ráez et al., 2010; Li et al., 2015). Ferulic acid inhibits mobility and is toxic to the burrowing nematode *R. similis* but is ineffective against *Pratylenchus penetrans* (Wuyts et al., 2006). Gallic acid acts as a nematicide to the root-knot nematode *M. incognita* (Seo et al., 2013). High constitutive total phenol contents were found in synthetic hexaploid wheat genotypes resistant to *P. thornei* combined with high levels of induced phenol oxidases (Rahaman et al., 2020). These studies indicate that the biochemical responses of host plants to both inoculation with AMF and infestation by plant-parasitic nematodes are highly complex.

Even within populations of a single species of AMF, there is a high genetic variability which may affect the host/fungal relationship (Koch et al., 2006, 2017). Variations in the effects that a single species of AMF have on *Pratylenchus* population densities were observed in the studies by Elsen et al. (2003b) and Jaizme-Vega and Pinochet (1997). Both studies used the same cultivar of banana and the same species of AMF, but obtained different results depending on the *Pratylenchus* sp. tested. Elsen et al. (2003b) stated that it was difficult to explain the contrary results, however, the AMF strain and the environmental conditions differed between experiments. As a different isolate of *F. mosseae* was used as inoculum, it is important to emphasize the traceability of isolates that are used in experiments. A similar observation was made in dune grass whereby *Pratylenchus* sp. were only reduced in the interaction with a community of AMF isolated from Wales and not from an AMF community isolated from Belgium (Rodríguez-Echeverría et al., 2009). This highlights the need to study interactions between specific crops, cultivars and AMF species or communities.

Plant functional group influenced *Pratylenchus* population densities in grasses but not in herbs and trees. Interestingly, response to AMF can be attributed to plant functional groups in which non-nitrogen fixing forbs and woody plants, and C4 grasses benefit more in plant growth by the fungal association, compared to nitrogen fixing plants and C3 grasses (Hoeksema et al., 2010). However, Yang et al. (2016) concluded that nitrogen fixing plants had a greater mycorrhizal growth response only when the host plant was a forb and not woody. A practical application to improve tolerance, or plant growth, when *Pratylenchus* is present may therefore be to pre-inoculate tree species with AMF prior to transplanting into orchards, taking into account the interaction between cultivars, their mycorrhizal dependency and AMF species used as inoculum sources (Pinochet et al., 1996). The potential of AMF inoculum conferring benefits to crop production in high economic value vegetable crops has been reviewed by Baum et al. (2015). These include advantages such as increases in yield, increases in commercial quality of the crop, protection against nematodes and other pathogens, tolerance to drought and other abiotic stressors and nutrient uptake. As the interaction between host, AMF inoculum and environment can be very specific, future research is needed to optimize the inoculation protocols to target specific crop production limitations.

The outcomes of the present systematic review, in relation to putative mechanisms involved in the interaction between *Pratylenchus* spp. and AMF, are discussed below.

### Enhanced Plant Tolerance

Plant shoot biomass increased when AMF were co-inoculated with *Pratylenchus* compared with infection with *Pratylenchus* alone. A number of studies investigated tolerance to *Pratylenchus* spp. as a reflection of increasing vegetative plant nutrition. AMF can increase the uptake of P and other nutrients such as Zn from the soil (Parniske, 2008; Seymour et al., 2019). This increase in nutrition can lead to a greater plant biomass response conferring a compensatory effect against the damage done by nematodes. Previous studies have shown that AMF confers tolerance to *Pratylenchus* spp. by compensating for root damage caused by *Pratylenchus* spp. through increasing the uptake of P and other micronutrients, such as Fe, Mn, Zn, and Cu (Calvet et al., 1995; Pinochet et al., 1998). However, improvement in the nutritional status of the plant is not believed to be wholly responsible for the biocontrol effect of AMF (Bødker et al., 1998; Jung et al., 2012).

Tolerance conferred by AMF to a crop under *Pratylenchus* pressure has been described in the majority of the reviewed papers (*n* = 41) with the exception of the following; peach, *Musa* sp., maize, tomato, dune grass and wheat (Pinochet et al., 1995b; Elsen et al., 2003a; Rodríguez-Echeverría et al., 2009; Vos et al., 2012; Brito et al., 2018; Frew et al., 2018). This may be a reflection of the mycorrhizal dependency of the cultivars assessed as some tomato and wheat cultivars have a low mycorrhizal dependency (Smith et al., 2009) while cultivars of maize, *Musa* sp. and peach generally have higher mycorrhizal dependency (Pinochet et al., 1995b; Kaeppler et al., 2000; Elsen et al., 2003a). A study by Martín-Robles et al. (2018) found that domesticated crops benefit more from the symbiosis with AMF under P limiting conditions. It is worthwhile to note that most of the studies analyzed in this review were undertaken in low P experimental conditions where AMF function most efficiently (Supplementary Table 1).

The studies assembled in Table 3 demonstrate the predominantly beneficial effects AMF have on crop species, alleviating the damage to the root and shoot biomass caused by *Pratylenchus*. There were only three studies where AMF decreased total biomass and root weight when co-inoculated with *Pratylenchus*. These studies were on wheat and dune grass, both C3 crops (Rodríguez-Echeverría et al., 2009; Frew et al., 2018). Variations in root morphology between C3 and C4 grasses determine their dependency on the mycorrhizal symbiosis (Hetrick et al., 1991), which may help explain the reduction in biomass. Wheat has a low to intermediate dependency on mycorrhiza depending on genotype (Lehnert et al., 2017) and modern plant breeding may contribute to a reduction in dependency on the mycorrhizal symbiosis by screening and selecting new varieties in high phosphate or highly fertile soils (Hetrick et al., 1993). However, a modern wheat cultivar Batavia was found to have high dependency on AMF colonization under drought conditions on a field site infested with *P. thornei* (Owen et al., 2010). Dune grass forms an association with AMF promoting plant growth (Tadych and Blaszkowski, 1999). de La Peña et al. (2006) suggested that evidence of biomass reduction in dune grass was related to a species-specific interaction between a geographically unique community of AMF from Wales and the species of *Pratylenchus* (*P. dunensis*) studied. Biomass reduction was not significant in another study of the interaction between AMF and *P. penetrans* on dune grass (de La Peña et al., 2006).

Previous reviews have also demonstrated this positive effect that AMF have on increasing plant growth under attack by migratory nematodes (Hol and Cook, 2005; Yang et al., 2014). This is contrary to the study by Borowicz (2001) that concluded AMF increased the negative effects of nematodes on plant biomass, indicating a reduced nematode tolerance.

The majority of studies showed an increase in root biomass in the presence of *Pratylenchus* when inoculated with AMF. *Pratylenchus* infestation negatively impacts root biomass, resulting in a reduction in the quantity and length of root branches (Fosu-Nyarko and Jones, 2016). Colonization by AMF can also result in alterations to root morphology, causing either an increase or decrease in root branching (Hooker et al., 1992; Sikes, 2010). A study on morphological changes within the root system in *Musa* sp. under *Pratylenchus* pressure showed that AMF increased root branching counteracting the negative consequences of *Pratylenchus* infection (Elsen et al., 2003a). Berta et al. (1995) also demonstrated in cherry plum (*Prunus cerasifera)* that AMF increased the branching of all root orders. However, there were variable effects on root diameter depending on which genera of AMF were used.

Baylis (1975) hypothesized that plants with extensive fine root systems with long dense root hairs were less reliant on the mycorrhizal symbiosis in comparison to coarsely rooted plants. However, recent evidence suggests that coarse roots are not necessarily a good predictor of crop dependency on the AMF symbiosis (Maherali, 2014). A meta-analysis by Yang et al. (2016) found that although plants with fibrous roots responded less to mycorrhizal colonization than tap rooted plant species, this was only evident for C3 and not C4 grass species. Notwithstanding this, plants that have a highly branched root system may still benefit from the AMF association via other ecosystem functions such as pathogen protection (Newsham et al., 1995).

### Competition for Space Between *Pratylenchus* and AMF

Degree of AMF colonization had an effect on the population densities of *Pratylenchus*. Inoculation with AMF that resulted in low levels of AMF colonization was associated with increases in *Pratylenchus* population densities compared with other cases with high levels of AMF colonization that were associated with decreases or no effects on *Pratylenchus* population densities. The nematode population density could also affect the rate of colonization by AMF indicating a competition between species. Both AMF and *Pratylenchus* occupy the same ecological niche within the root cortical cells as described in various crop species, for example, quince, cherry, peach, pear, banana, plum, and coffee (Calvet et al., 1995; Pinochet et al., 1995a,b, 1998; Lopez et al., 1997; Vaast et al., 1997; Elsen et al., 2003b). *Pratylenchus* sp. and AMF were considered to have competed for space within the cortical cells in quince, coffee, banana and dune grass (Calvet et al., 1995; Vaast et al., 1997; Elsen et al., 2003b; de La Peña et al., 2006).

Arbuscules are the metabolically active sites of exchange between the plant and the fungus and a mature mycorrhizal colonization of the plant, as evidenced by the production of arbuscules, has been thought to be the prerequisite for a biocontrol effect (Khaosaad et al., 2007). It has been hypothesized that a greater colonization of AMF in plant roots would lead to a greater biocontrol effect on nematodes.

*Pratylenchus* can affect the quantity and morphology of AMF within the root cortical cells. For example, in quince, AMF increased the production of arbuscules reflecting a metabolically active state under *Pratylenchus* infestation, compared to an increase in the production of vesicles in the absence of infestation (Calvet et al., 1995). In banana, nematodes reduced the frequency of colonization but not the intensity (Elsen et al., 2003b). In pineapple, although nematodes reduced the frequency of arbuscules when applied at a later time point during transplanting, they did not affect the efficiency of the symbiosis (Guillemin et al., 1994).

The time of inoculation was not a factor in how the nematode population densities responded to AMF inoculation. AMF was applied to the plants prior to nematode inoculation in the majority of studies (*n* = 42), which gave the symbiosis a chance to establish before being challenged with *Pratylenchus*. However, this established symbiosis was not reflected in a decrease in nematode population density, but may have aided the plant in tolerance to nematode infestation through increased vegetative growth as previously discussed.

### Plant Defense and Induced Systemic Resistance

Mycorrhiza-induced resistance that can operate systemically can be effective against plant-parasitic nematodes and may contribute toward the biocontrol effect of AMF (Jung et al., 2012). Induced systemic resistance has no association with pathogenesis related proteins or salicylic acid but is regulated by jasmonic acids and ethylene (Pieterse et al., 1998).

There is little available research on induced systemic resistance by AMF against *Pratylenchus* as compared to other plant pathogens. However, using split root experiments, the systemic biocontrol effects of the AMF species *F. mosseae* and *R. irregularis* on *Pratylenchus* were demonstrated in banana and tomato. *Rhizophagus irregularis* induced a systemic suppression of *P. coffeae* and *R. similis* in banana, though the pathways involved in this suppression were not determined (Elsen et al., 2008). In tomato, inoculation with *F. mosseae* reduced the number of females of *P. penetrans* through a localized mechanism and the number of juveniles through a systemic mechanism (Vos et al., 2012). Contrary to this, only a localized suppression of *Pratylenchus* population densities was observed in dune grass (de La Peña et al., 2006).

Investigations into the metabolomics of AMF showed that AMF colonization increased the production of AMF plant signaling compounds and anti-herbivory defenses (Hill et al., 2018). There is still very little research available on the interactions between *Pratylenchus* and AMF on effects on the metabolome. Frew et al. (2018) reported that AMF reduced plant defense metabolites, specifically benzoxazinoids, which accounted for an increase in *P. neglectus* population densities in wheat. Studies involving root organ cultures of carrot showed significant suppressive effects of AMF on *P. coffeae* female population densities believed to be a result of biochemical changes in the mycorrhized root (Elsen et al., 2003c). Exudates from AMF can reduce the motility and penetration of sedentary nematodes (Vos et al., 2012) but little research has been done on their effects on migratory endo-parasites. An *in-vitro* chemotaxic assay on the migratory endo-parasite *R. similis* demonstrated that the exudation of a water-soluble compound, produced by mycorrhizal roots, reduced attraction at a pre-infection stage (Vos et al., 2012), but there is little information on how exudates affect *Pratylenchus* spp. Further research is needed to assess the mechanisms of AMF in influencing *Pratylenchus* population densities.

### Alterations in the Rhizosphere

Alterations in chemical compounds in the rhizosphere as a result of interactions between plant-parasitic nematodes and AMF have been reviewed (Schouteden et al., 2015). These involve changes in exudation of sugars, organic acids, amino acids, phenolic compounds, flavonoids and strigolactones in AMF colonized plants as compared to non-AMF plants. AMF exudations into the rhizosphere promote beneficial microorganisms such as plant-growth promoting rhizobacteria (PGPR) (Jung et al., 2012; Javaid, 2017) and resultant changes can be induced systemically, influencing the bacterial community structure (Marschner and Baumann, 2003). This enhanced microbial activity around plant roots has been termed the mycorrhizosphere effect (Linderman, 1988). Plant growth promoting rhizobacteria have been implicated in nitrogen fixation, phosphate solubilization, modulating phytohormone levels and the production of antibiotics and lytic enzymes (Glick, 2012). Cameron et al. (2013) proposed that AMF and PGPR act together to increase plant defenses against biotic stressors in mycorrhiza-induced resistance. Studies on multipartite interactions between *Pratylenchus*, AMF, PGPR and crop hosts are lacking in the literature.

Species of PGPR in the genera *Pseudomonas, Bacillus, Streptomyces* and *Lysobacter* have been implicated in reducing *Pratylenchus* population densities (Walker et al., 1966; Stirling, 2014; Castillo et al., 2017), and some research has been conducted on the interaction between AMF and these PGPR. In strawberry, *Pseudomonas chlororaphis* suppressed populations of *P. penetrans* (Hackenberg et al., 2000) while extracts from the AMF species *R. irregularis* stimulated the growth of *Pseudomonas chlororaphis in vitro* (Filion et al., 1999). *Streptomyces* spp. can reduce *Pratylenchus* population densities (Meyer and Linderman, 1986; Samac and Kinkel, 2001) and they can also stimulate spore germination in *F. mosseae* and *Gigaspora margarita* (Tylka et al., 1991). This indicates a link between the three types of phytobiome organisms, though further research is needed to assess AMF and PGPR combined effects on *Pratylenchus* population densities.

## Limitations of the Review and Future Research

The crops assessed in this review were agriculturally or horticulturally important with the exception of dune grass (*Ammophilia arenaria*). Most studies looked at a single species of AMF alone and not in combination with species from different orders and genera of AMF, or other beneficial microbes such as PGPR. The taxonomic orders of AMF used in the studies reviewed were limited to the Glomerales and Diversisporales. Other orders such as the Archaeosporales and the Paraglomerales are also present in soils, though they are under-represented in experimental work. A study by Gosling et al. (2014), found a wide distribution of the Paraglomerales in agricultural soils in the UK. AMF species such as *F. mosseae* and *R. irregularis* have a tendency to be over represented in this type of experimental work due to their ease of multiplication in trap cultures. The studies in this review were undertaken in low P soils, predominantly in glasshouses, with some transplantations to microplots. Arbuscular mycorrhizal fungi function most efficiently under low to moderately high P conditions, and therefore the benefit of AMF in improving plant nutrition and plant biomass under *Pratylenchus* pressure could be overstated for agricultural systems receiving continued high rates of P fertilizers. Better matching of P fertilizer inputs to crop removal is required in some agricultural systems to avoid excessive levels of available P in soils for better harnessing of AMF functions, stewardship of global P supplies and environmental quality (Gianinazzi et al., 2010).

The number of studies in this highly specific review of the interaction between *Pratylenchus* spp. and AMF was limited to only 60 studies suitable for inclusion. Further research needs to be undertaken in the area, using a broad range of crop cultivars and AMF species from diverse orders to further increase our understanding of the relationship between these organisms in the rhizosphere.

Further research needs to be done in assessing the mechanisms involved in the effect of AMF on *Pratylenchus* population densities through investigations into induced systemic resistance and changes in the metabolome. As research is lacking on the effects of AMF, *Pratylenchus* and beneficial bacteria in the rhizosphere, more studies need to be undertaken on multipartite interactions between these organisms in crop hosts.

## Conclusion

The interactions between *Pratylenchus* and AMF reveal some unique effects as influenced by crop species, crop cultivar, AMF order and AMF genus. Our review showed increased *Pratylenchus* densities in plants inoculated with species from the order Diversisporales. Inoculation with the AMF genera *Glomus* and *Funneliformis* from the order Glomerales, reduced or had no effect on *Pratylenchus* densities in host roots. AMF aids the tolerance of plants to *Pratylenchus* through increased vegetative growth. The biocontrol effect of AMF is likely to be a combination of increasing host tolerance, competition between organisms, and systemic resistance, though further research is needed to identify the mechanisms involved. Further studies will need to take into account the specific interactions between crop, cultivar and AMF species in both glasshouse and field trials.

## Data Availability Statement

All datasets generated for this study are included in the article/Supplementary Material.

## Author Contributions

JT and EG conceptualized the paper. EG performed the database search, collated the data, and drafted the manuscript. JT and EG conducted the statistical analyses. EG, KO, and RZ integrated information on tables. All authors contributed to revising the manuscript. All authors contributed to the article and approved the submitted version.

## Conflict of Interest

The authors declare that the research was conducted in the absence of any commercial or financial relationships that could be construed as a potential conflict of interest.

## References

[B1] Baum C. El-Tohamy W. Gruda N. (2015). Increasing the productivity and product quality of vegetable crops using arbuscular mycorrhizal fungi: a review. Sci. Hortic. 187, 131–141. 10.1016/j.scienta.2015.03.002

[B2] Baylis G. T. S. (1975). The magnolioid mycorrhiza and mycotrophy in root systems derived from it. in: Endomycorrhizas, eds Sanders F.E. Mosse B. Tinker P.B. (London, UK: Academic Press), 373–389.

[B3] Berruti A. Lumini E. Balestrini R. Bianciotto V. (2016). Arbuscular mycorrhizal fungi as natural biofertilizers: let's benefit from past successes. Front. Microbiol. 6:1559. 10.3389/fmicb.2015.01559 26834714 PMC4717633

[B4] Berta G. Trotta A. Fusconi A. Hooker J. E. Munro M. Atkinson D.. (1995). Arbuscular mycorrhizal induced changes to plant growth and root system morphology in *Prunus cerasifera*. Tree Physiol. 15, 281–293. 10.1093/treephys/15.5.281 14965952

[B5] Bødker L. Kjøller R. Rosendahl S. (1998). Effect of phosphate and the arbuscular mycorrhizal fungus *Glomus intraradices* on disease severity of root rot of peas (*Pisum sativum*) caused by *Aphanomyces euteiches*. Mycorrhiza 8, 169–174. 10.1007/s005720050230

[B6] Borowicz V. A. (2001). Do arbuscular mycorrhizal fungi alter plant-pathogen relations? Ecology 82, 3057–3068. 10.1890/0012-9658(2001)082[3057:DAMFAP]2.0.CO;2

[B7] Brito O. D. C. Hernandes I. Ferreira J. C. A. Cardoso M. R. Alberton O. Dias-Arieira C. R. (2018). Association between arbuscular mycorrhizal fungi and *Pratylenchus brachyurus* in maize crop. Chilean J. Agric. Res. 78, 521–527. 10.4067/S0718-58392018000400521

[B8] Calvet C. Pinochet J. Camprub,í A. Fernández C. (1995). Increased tolerance to the root-lesion nematode *Pratylenchus vulnus* in mycorrhizal micropropagated BA-29 quince rootstock. Mycorrhiza 5, 253–258. 10.1007/BF00204958

[B9] Cameron D. D. Neal A. L. van Wees S. C. M. Ton J. (2013). Mycorrhiza-induced resistance: more than the sum of its parts? Trends Plant Sci. 18, 539–545. 10.1016/j.tplants.2013.06.004 23871659 PMC4194313

[B10] Camprubi A. Pinochet J. Calvet C. Estaun V. (1993). Effects of the root-lesion nematode *Pratylenchus vulnus* and the vesicular-arbuscular mycorrhizal fungus *Glomus mosseae* on the growth of three plum rootstocks. Plant Soil 153, 223–229. 10.1007/BF00012995

[B11] Castillo J. D. Vivanco J. M. Manter D. K. (2017). Bacterial microbiome and nematode occurrence in different potato agricultural soils. Microb. Ecol. 74, 888–900. 10.1007/s00248-017-0990-2 28528399

[B12] Castillo P. Vovlas N. (2007). Pratylenchus (Nematoda: Pratylenchidae): diagnosis, biology, pathogenicity and management, in Nematology Monographs and Perspectives, Vol. 6, eds Hunt D. J Perry R. N (Brill: Leiden), 1–7. 10.1163/ej.9789004155640.i-523

[B13] Ceustermans A. van Hemelrijck W. Van Campenhout J. Bylemans D. (2018). Effect of arbuscular mycorrhizal fungi on *Pratylenchus penetrans* infestation in apple seedlings under greenhouse conditions. Pathogens 7:76. 10.3390/pathogens7040076 30241406 PMC6313298

[B14] de La Peña E. Echeverría S. R. van der Putten W. H. Freitas H. Moens M. (2006). Mechanism of control of root-feeding nematodes by mycorrhizal fungi in the dune grass *Ammophila arenaria*. N. Phytol. 169, 829–840. 10.1111/j.1469-8137.2005.01602.x 16441763

[B15] de Luca F. Reyes A. Troccoli A. Castillo P. (2011). Molecular variability and phylogenetic relationships among different species and populations of *Pratylenchus* (Nematoda: Pratylenchidae) as inferred from the analysis of the ITS rDNA. Eur. J. Plant Pathol. 130, 415–426. 10.1007/s10658-011-9763-9

[B16] Decraemer W. Hunt D. J. (2013). Structure and classification, in: *Plant Nematology, 2*^*nd*^ *Edn*, eds Perry R. N. Hunt D. J. (Wallingford: CAB International), 3–39. 10.1079/9781780641515.0003

[B17] Elliott A. Bird G. Safir G. (1984). Joint influence of *Pratylenchus penetrans* (Nematoda) and *Glomus fasciculatum* (Phycomyceta) on the ontogeny of *Phaseolus vulgaris*. Nematropica 14, 111–119.

[B18] Elsen A. Baimey H. Swennen R. de Waele D. (2003b). Relative mycorrhizal dependency and mycorrhiza-nematode interaction in banana cultivars (*Musa* spp.) differing in nematode susceptibility. Plant Soil 256, 303–313. 10.1023/A:1026150917522

[B19] Elsen A. Beeterens R. Swennen R. de Waele D. (2003a). Effects of an arbuscular mycorrhizal fungus and two plant-parasitic nematodes on *Musa* genotypes differing in root morphology. Biol. Fertil. Soils 38, 367–376. 10.1007/s00374-003-0669-3

[B20] Elsen A. Declerck S. Waele D. (2003c). Use of root organ cultures to investigate the interaction between *Glomus intraradices* and *Pratylenchus coffeae*. Appl. Environ. Microbiol. 69, 4308–4311. 10.1128/AEM.69.7.4308-4311.2003 12839820 PMC165172

[B21] Elsen A. Gervacio D. Swennen R. de Waele D. (2008). AMF-induced biocontrol against plant parasitic nematodes in *Musa* sp.: a systemic effect. Mycorrhiza 18, 251–256. 10.1007/s00572-008-0173-6 18392645

[B22] Filion M. St-Arnaud M. Fortin J. (1999). Direct interaction between the arbuscular mycorrhizal fungus *Glomus intraradices* and different rhizosphere microorganisms. N. Phytol. 141, 525–533. 10.1046/j.1469-8137.1999.00366.x

[B23] Forge T. Muehlchen A. Hackenberg C. Neilsen G. Vrain T. (2001). Effects of preplant inoculation of apple (*Malus domestica* Borkh.) with arbuscular mycorrhizal fungi on population growth of the root-lesion nematode, *Pratylenchus penetrans*. Plant Soil 236, 185–196. 10.1023/A:1012743028974

[B24] Fosu-Nyarko J. Jones M. G. (2016). Advances in understanding the molecular mechanisms of root lesion nematode host interactions. Annu. Rev. Phytopathol. 54, 253–278. 10.1146/annurev-phyto-080615-100257 27296144

[B25] Frew A. Powell J. R. Glauser G. Bennett A. E. Johnson S. N. (2018). Mycorrhizal fungi enhance nutrient uptake but disarm defences in plant roots, promoting plant-parasitic nematode populations. Soil Biol. Biochem. 126, 123–132. 10.1016/j.soilbio.2018.08.019

[B26] Gianinazzi S. Gollote A. Binet M. N. van Tuinen D. Redecker D. Wipf D. (2010). Agroecology: the key role of arbuscular mycorrhizas in ecosystem services. Mycorrhiza 20, 519–530. 10.1007/s00572-010-0333-3 20697748

[B27] Glick B. R. (2012). Plant growth-promoting bacteria: mechanisms and applications. Scientifica 2012:963401. 10.6064/2012/963401 24278762 PMC3820493

[B28] Gosling P. Proctor M. Jones J. Bending G. D. (2014). Distribution and diversity of *Paraglomus* spp. in tilled agricultural soils. Mycorrhiza 24, 1–11. 10.1007/s00572-013-0505-z 23715868

[B29] Guillemin J.-P. Gianinazzi S. Gianinazzi-Pearson V. Marchal J. (1994). Control by arbuscular endomycorrhizae of *Pratylenchus brachyurus* in pineapple microplants. Agric. Food Sci. 3, 253–262. 10.23986/afsci.72703

[B30] Hackenberg C. Muehlkchen A. Forge T. Vrain T. (2000). *Pseudomonas chlororaphis* strain Sm3, bacterial antagonist of *Pratylenchus penetrans*. J. Nematol. 32, 183–189. 19270964 PMC2620438

[B31] Hart M. M. Reader R. J. (2002). Taxonomic basis for variation in the colonization strategy of arbuscular mycorrhizal fungi. N. Phytol. 153, 335–344. 10.1046/j.0028-646X.2001.00312.x

[B32] Hetrick B. A. D. Wilson G. W. T. Cox T. S. (1993). Mycorrhizal dependence of modern wheat cultivars and ancestors: a synthesis. Can. J. Bot. 71, 512–518. 10.1139/b93-056

[B33] Hetrick B. A. D. Wilson G. W. T. Leslie J. F. (1991). Root architecture of warm-and cool-season grasses: relationship to mycorrhizal dependence. Can. J. Bot. 69, 112–118. 10.1139/b91-016

[B34] Hill E. M. Robinson L. A. Abdul-Sada A. Vanbergen A. J. Hodge A. Hartley S. E. (2018). Arbuscular mycorrhizal fungi and plant chemical defence: effects of colonisation on aboveground and belowground metabolomes. J. Chem. Ecol. 44, 198–208. 10.1007/s10886-017-0921-1 29392532 PMC5843688

[B35] Hoeksema J. D. Chaudhary V. B. Gehring C. A. Johnson N. C. Karst J. Koide R. T.. (2010). A meta-analysis of context-dependency in plant response to inoculation with mycorrhizal fungi. Ecol. Lett. 13, 394–407. 10.1111/j.1461-0248.2009.01430.x 20100237

[B36] Hol W. H. G. Cook R. (2005). An overview of arbuscular mycorrhizal fungi–nematode interactions. Basic Appl. Ecol. 6, 489–503. 10.1016/j.baae.2005.04.001

[B37] Hooker J. Munro M. Atkinson D. (1992). Vesicular-arbuscular mycorrhizal fungi induced alteration in poplar root system morphology. Plant Soil 145, 207–214. 10.1007/BF00010349

[B38] Hussey R. Roncadori R. (1978). Interaction of *Pratylenchus brachyurus* and *Gigaspora margarita* on cotton. J. Nematol. 10, 16–20. 19305806 PMC2617853

[B39] Jaizme-Vega M. Pinochet J. (1997). Growth response of banana to three mycorrhizal fungi in *Pratylenchus goodeyi* infested soil. Nematropica 27, 69–76. 10.1023/A:1004236310644

[B40] Javaid A. (2017). Role of AMF in nitrogen fixation in legumes, in Microbes for Legume Improvement, eds Zaidi A. Khan M. S. Musarrat J. (Cham: Springer International Publishing), 409–426.

[B41] Jones J. T. Haegeman A. Danchin E. G. J. Gaur H. S. Helder J. Jones M. G. K.. (2013). Top 10 plant-parasitic nematodes in molecular plant pathology. Mol. Plant Pathol. 14, 946–961. 10.1111/mpp.12057 23809086 PMC6638764

[B42] Jones M. G. K. Fosu-Nyarko J. (2014). Molecular biology of root lesion nematodes (*Pratylenchus* spp.) and their interaction with host plants: molecular biology of root lesion nematodes. Ann. Appl. Biol. 164, 163–181. 10.1111/aab.12105

[B43] Jung S. C. Martinez-Medina A. Lopez-Raez J. A. Pozo M. J. (2012). Mycorrhiza-induced resistance and priming of plant defenses. J. Chem. Ecol. 38, 651–664. 10.1007/s10886-012-0134-6 22623151

[B44] Kaeppler S. M. Parke J. L. Mueller S. M. Senior L. Stuber C. Tracy W. F. (2000). Variation among maize inbred lines and detection of quantitative trait loci for growth at low phosphorus and responsiveness to arbuscular mycorrhizal fungi. Crop Sci. 40, 358–364. 10.2135/cropsci2000.402358x

[B45] Khaosaad T. Garcia-Garrido J. Steinkellner S. Vierheilig H. (2007). Take-all disease is systemically reduced in roots of mycorrhizal barley plants. Soil Biol. Biochem. 39, 727–734. 10.1016/j.soilbio.2006.09.014

[B46] Klironomos J. N. (2000). Host-specificity and functional diversity among arbuscular mycorrhizal fungi, in Microbial Biosystems: New Frontiers. Proceedings of the Eighth International Symposium on Microbial Ecology, eds Bell C. R. Brylinsky M. Johnson-Green P. (Halifax: Atlantic Canada Society for Microbial Ecology), 845–851.

[B47] Koch A. M. Antunes P. M. Maherali H. Hart M. M. Klironomos J. N. (2017). Evolutionary asymmetry in the arbuscular mycorrhizal symbiosis: conservatism in fungal morphology does not predict host plant growth. N. Phytol. 214, 1330–1337. 10.1111/nph.14465 28186629

[B48] Koch A. M. Croll D. Sanders I. R. (2006). Genetic variability in a population of arbuscular mycorrhizal fungi causes variation in plant growth. Ecol. Lett. 9, 103–110. 10.1111/j.1461-0248.2005.00853.x 16958874

[B49] Lambers H. Teste F. P. (2013). Interactions between arbuscular mycorrhizal and non-mycorrhizal plants: do non-mycorrhizal species at both extremes of nutrient availability play the same game? Plant Cell Environ. 36, 1911–1915. 10.1111/pce.12117 23586707

[B50] Lehnert H. Serfling A. Enders M. Friedt W. Ordon F. (2017). Genetics of mycorrhizal symbiosis in winter wheat (*Triticum aestivum*). N. Phytol. 215, 779–791. 10.1111/nph.14595 28517039

[B51] Leifheit E. F. Veresoglou S. D. Lehmann A. Morris E. K. Rillig M. C. (2014). Multiple factors influence the role of arbuscular mycorrhizal fungi in soil aggregation—a meta-analysis. Plant Soil 374, 523–537. 10.1007/s11104-013-1899-2

[B52] Li J. F. He X. H. Li H. Zheng W. J. Liu J. F. Wang M. Y. (2015). Arbuscular mycorrhizal fungi increase growth and phenolics synthesis in *Poncirus trifoliata* under iron deficiency. Sci. Hortic. 183, 87–92. 10.1016/j.scienta.2014.12.015

[B53] Linderman R. (1988). Mycorrhizal interactions with the rhizosphere microflora: the mycorrhizosphere effect. Phytopathology 78, 366–371.

[B54] Lopez A. Pinochet J. Fernandez C. Calvet C. Camprubi A. (1997). Growth response of OHF-333 pear rootstock to arbuscular mycorrhizal fungi, phosphorus nutrition and *Pratylenchus vulnus* infection. Fundam. Appl. Nematol. 20, 87–93.

[B55] López-Ráez J. A. Flors V. García J. M. Pozo M. J. (2010). AM symbiosis alters phenolic acid content in tomato roots. Plant Signal. Behav. 5, 1138–1140. 10.4161/psb.5.9.12659 21490421 PMC3115087

[B56] Maherali H. (2014). Is there an association between root architecture and mycorrhizal growth response? New Phytol. 204, 192–200. 10.1111/nph.12927 25041241

[B57] Maherali H. Klironomos J. N. (2007). Influence of phylogeny on fungal community assembly and ecosystem functioning. Science 316, 1746–1748. 10.1126/science.1143082 17588930

[B58] Marschner P. Baumann K. (2003). Changes in bacterial community structure induced by mycorrhizal colonisation in split-root maize. Plant Soil 251, 279–289. 10.1023/A:1023034825871

[B59] Martín-Robles N. Lehmann A. Seco E. Aroca R. Rillig M. C. Milla R. (2018). Impacts of domestication on the arbuscular mycorrhizal symbiosis of 27 crop species. N. Phytol. 218, 322–334. 10.1111/nph.14962 29281758

[B60] Meyer J. R. Linderman R. (1986). Selective influence on populations of rhizosphere or rhizoplane bacteria and actinomycetes by mycorrhizas formed by *Glomus fasciculatum*. Soil Biol. Biochem. 18, 191–196. 10.1016/0038-0717(86)90026-X

[B61] Moher D. Liberati A. Tetzlaff J. Altman D. G. (2009). Preferred reporting items for systematic reviews and meta-analyses: the PRISMA statement. Ann. Intern. Med. 151, 264–269. 10.7326/0003-4819-151-4-200908180-00135 19622511

[B62] Morton J. B. Benny G. L. (1990). Revised classification of arbuscular mycorrhizal fungi (Zygomycetes): a new order Glomales, two new suborders, Glomineae and Gigasporineae and two new families, Acaulosporaceae and Gigasporaceae, with an emendation to Glomaceae. Mycotaxon 37, 471–491.

[B63] Newsham K. K. Fitter A. H. Watkinson A. R. (1995). Multi-functionality and biodiversity in arbuscular mycorrhizas. Trends Ecol. Evol. 10, 407–411. 10.1016/S0169-5347(00)89157-0 21237085

[B64] Owen K. J. Clewett T. G. Thompson J. P. (2010). Pre-cropping with canola decreased *Pratylenchus thornei* populations, arbuscular mycorrhizal fungi, and yield of wheat. Crop Pasture Sci. 61, 399–410. 10.1071/CP09345

[B65] Parniske M. (2008). Arbuscular mycorrhiza: the mother of plant root endosymbioses. Nat. Rev. Microbiol. 6, 763–775. 10.1038/nrmicro1987 18794914

[B66] Pieterse C. M. van Wees S. C. van Pelt J. A. Knoester M. Laan R. Gerrits H.. (1998). A novel signaling pathway controlling induced systemic resistance in *Arabidopsis*. Plant Cell 10, 1571–1580. 10.1105/tpc.10.9.1571 9724702 PMC144073

[B67] Pinochet J. Calvet C. Camprubi A. Fernandez C. (1995a). Growth and nutritional response of Nemared peach rootstock infected with *Pratylenchus vulnus* and the mycorrhizal fungus *Glomus mosseae*. Fundam. Appl. Nematol. 18, 205–210.

[B68] Pinochet J. Calvet C. Camprubi A. Fernández C. (1995b). Interaction between the root-lesion nematode *Pratylenchus vulnus* and the mycorrhizal association of *Glomus intraradices* and Santa Lucia 64 cherry rootstock. Plant Soil 170, 323–329. 10.1007/BF00010485

[B69] Pinochet J. Calvet C. Camprubí A. Fernández C. (1996). Interactions between migratory endoparasitic nematodes and arbuscular mycorrhizal fungi in perennial crops: a review. Plant Soil 185, 183–190. 10.1007/BF02257523

[B70] Pinochet J. Camprubi A. Calvet C. (1993). Effects of the root-lesion nematode *Pratylenchus vulnus* and the mycorrhizal fungus *Glomus mosseae* on the growth of EMLA-26 apple rootstock. Mycorrhiza 4, 79–83. 10.1007/BF00204062

[B71] Pinochet J. Camprubi A. Calvet C. Fernandez C. Kabana R. R. (1998). Inducing tolerance to the root-lesion nematode *Pratylenchus vulnus* by early mycorrhizal inoculation of micropropagated Myrobalan 29 C plum rootstock. J. Am. Soc. Horti. Sci. 123:342 10.21273/JASHS.123.3.342

[B72] Powell J. R. Rillig M. C. (2018). Biodiversity of arbuscular mycorrhizal fungi and ecosystem function. N. Phytol. 220, 1059–1075. 10.1111/nph.15119 29603232

[B73] Pozo M. J. Azcón-Aguilar C. (2007). Unraveling mycorrhiza-induced resistance. Curr. Opin. Plant Biol. 10, 393–398. 10.1016/j.pbi.2007.05.004 17658291

[B74] Pozo M. J. Cordier C. Dumas-Gaudot E. Gianinazzi S. Barea J. M. Azcón-Aguilar C. (2002). Localized versus systemic effect of arbuscular mycorrhizal fungi on defence responses to *Phytophthora infection* in tomato plants. J. Exp. Bot. 53, 525–534. 10.1093/jexbot/53.368.525 11847251

[B75] Rahaman M. M. Zwart R. S. Thompson J. P. (2020). Constitutive and induced expression of total phenol and phenol oxidases in wheat genotypes ranging in resistance/susceptibility to the root-lesion nematode *Pratylenchus thornei*. Plants 9:485. 10.3390/plants9040485 32283872 PMC7238097

[B76] Redecker D. Schüßler A. Stockinger H. Stürmer S. L. Morton J. B. Walker C. (2013). An evidence-based consensus for the classification of arbuscular mycorrhizal fungi (Glomeromycota). Mycorrhiza 23, 515–531. 10.1007/s00572-013-0486-y 23558516

[B77] Rivero J. Gamir J. Aroca R. Pozo M. J. Flors V. (2015). Metabolic transition in mycorrhizal tomato roots. Front. Microbiol. 6:598. 10.3389/fmicb.2015.00598 26157423 PMC4477175

[B78] Rodríguez-Echeverría S. de La Peña E. Moens M. Freitas H. Van Der Putten W. H. (2009). Can root-feeders alter the composition of AMF communities? Experimental evidence from the dune grass *Ammophila arenaria*. Basic Appl. Ecol. 10, 131–140. 10.1016/j.baae.2008.01.004

[B79] Samac D. A. Kinkel L. L. (2001). Suppression of the root-lesion nematode (*Pratylenchus penetrans*) in alfalfa (*Medicago sativa*) by *Streptomyces* spp. Plant Soil 235, 35–44. 10.1023/A:1011820002779

[B80] Schouteden N. de Waele D. Panis B. Vos C. M. (2015). Arbuscular mycorrhizal fungi for the biocontrol of plant-parasitic nematodes: a review of the mechanisms involved. Front. Microbiol. 6:1280. 10.3389/fmicb.2015.01280 26635750 PMC4646980

[B81] Schüßler A. Walker C. (2010). The Glomeromycota: A Species List With New Families and New Genera. The Royal Botanic Garden Kew, Botanische Staatssammlung Munich, and Oregon State University.

[B82] Seo D. J. Nguyen V. N. Kim K. Y. Park R. D. Jung W. J. (2013). Nematicidal activity of gallic acid purified from *Terminalia nigrovenulosa* bark against the root-knot nematode *Meloidogyne incognita*. Nematology 15, 507–518. 10.1163/15685411-00002696

[B83] Seymour N. P. Edwards D. G. Thompson J. P. (2019). A dual rescaled Mitscherlich model of the simultaneous savings in phosphorus and zinc fertiliser from arbuscular mycorrhizal fungal colonisation of linseed (*Linum usitatissimum* L). Plant Soil 440, 97–118. 10.1007/s11104-019-04065-2

[B84] Sidker M. M. Vestergård M. (2019). Impacts of root metabolites on soil nematodes. Front. Plant Sci. 10:1792. 10.3389/fpls.2019.01792 32082349 PMC7005220

[B85] Sikes B. A. (2010). When do arbuscular mycorrhizal fungi protect plant roots from pathogens? Plant Signal. Behav. 5, 763–765. 10.4161/psb.5.6.11776 20400855 PMC3001584

[B86] Singh S. K. Hodda M. Ash G. J. Banks N. C. (2013). Plant-parasitic nematodes as invasive species: characteristics, uncertainty and biosecurity implications. Ann. Appl. Biol. 163, 323–350. 10.1111/aab.12065

[B87] Smith F. A. Grace E. J. Smith S. E. (2009). More than a carbon economy: nutrient trade and ecological sustainability in facultative arbuscular mycorrhizal symbioses. N. Phytol. 182, 347–358. 10.1111/j.1469-8137.2008.02753.x 19207688

[B88] Smith S. E. Jakobsen I. Grønlund M. Smith A. (2011). Roles of arbuscular mycorrhizas in plant phosphorus nutrition: interactions between pathways of phosphorus uptake in arbuscular mycorrhizal roots have important implications for understanding and manipulating plant phosphorus acquisition. Plant Physiol. 156, 1050–1057. 10.1104/pp.111.174581 21467213 PMC3135927

[B89] Smith S. E. Read D. J. (2007). Mycorrhizal Symbiosis. San Diego, CA: Elsevier Science.

[B90] Smith S. E. Read D. J. (2008). Mycorrhizal symbiosis. Amsterdam, Boston, MA: Academic Press.

[B91] Smith S. E. Smith F. A. Jakobsen I. (2004). Functional diversity in arbuscular mycorrhizal (AM) symbioses: the contribution of the mycorrhizal P uptake pathway is not correlated with mycorrhizal responses in growth or total P uptake. New Phytol. 162, 511–524. 10.1111/j.1469-8137.2004.01039.x

[B92] Soriano I. Asenstorfer R. Schmidt O. Riley I. (2004). Inducible flavone in oats (*Avena sativa*) is a novel defense against plant-parasitic nematodes. Phytopathology 94, 1207–1214. 10.1094/PHYTO.2004.94.11.1207 18944456

[B93] Steel G. D. Torrie J. H. (1960). Principles and Procedures of Statistics. New York, NY: McGraw Hill 19, 366–387.

[B94] Stirling G. R. (2014). Biological Control of Plant-Parasitic Nematodes: Soil Ecosystem Management in Sustainable Agriculture. Wallingford: CABI.

[B95] Tadych M. Blaszkowski J. (1999). Growth responses of maritime sand dune plant species to arbuscular mycorrhizal fungi. Acta Mycol. 34, 115–124. 10.5586/am.1999.010

[B96] Talavera M. Itou K. Mizukubo T. (2001). Reduction of nematode damage by root colonization with arbuscular mycorrhiza (*Glomus* spp.) in tomato-*Meloidogyne incognita* (Tylenchida: *Meloidogynidae*) and carrot-*Pratylenchus penetrans* (Tylenchida: *Pratylenchidae*) pathosystems. Appl. Entomol. Zool. 36, 387–392. 10.1303/aez.2001.387

[B97] Thompson J. P. (1993). What is the potential for management of mycorrhizas in agriculture?, in: Management of Mycorrhizas in Agriculture, Horticulture and Forestry, Proceedings of an International Symposium on Management of Mycorrhizas in Agriculture, Horticulture and Forestry (Perth, WA, Australia), eds Robson A. D. Abbott L. K. Malajczuk N. (Dordecht: Kluwer Academic Publishers), 191–200.

[B98] Tylka G. Hussey R. Roncadori R. (1991). Axenic germination of vesicular-arbuscular mycorrhizal fungi: effects of selected *Streptomyces* species. Phytopathology 81, 754–759. 10.1094/Phyto-81-754

[B99] Vaast P. Caswell-Chen E. P. Zasoski R. J. (1997). Influences of a root-lesion nematode, *Pratylenchus coffeae*, and two arbuscular mycorrhizal fungi, *Acaulospora mellea* and *Glomus clarum* on coffee (*Coffea arabica* L.). Biol. Fertil. Soils 26, 130–135. 10.1007/s003740050355

[B100] Veresoglou S. D. Rillig M. C. (2012). Suppression of fungal and nematode plant pathogens through arbuscular mycorrhizal fungi. Biol. Lett. 8, 214–217. 10.1098/rsbl.2011.0874 22012951 PMC3297398

[B101] Vos C. M. Tesfahun A. N. Panis B. de Waele D. Elsen A. (2012). Arbuscular mycorrhizal fungi induce systemic resistance in tomato against the sedentary nematode *Meloidogyne incognita* and the migratory nematode *Pratylenchus penetrans*. Appl. Soil Ecol. 61, 1–6. 10.1016/j.apsoil.2012.04.007

[B102] VSN International (2014). GenStat for Windows, 17th Edn, Hemel Hempstead: VSN International.

[B103] Walker J. Specht C. Bekker J. (1966). Nematocidal activity to *Pratylenchus penetrans* by culture fluids from actinomycetes and bacteria. Can. J. Microbiol. 12, 347–351. 10.1139/m66-047 5951708

[B104] Whipps J. M. (2004). Prospects and limitations for mycorrhizas in biocontrol of root pathogens. Can. J. Bot. 82, 1198–1227. 10.1139/b04-082

[B105] Wuyts N. Swennen R. de Waele D. (2006). Effects of plant phenylpropanoid pathway products and selected terpenoids and alkaloids on the behaviour of the plant-parasitic nematodes *Radopholus similis, Pratylenchus penetrans* and *Meloidogyne incognita*. Nematology 8, 89–101. 10.1163/156854106776179953

[B106] Yang H. Dai Y. Wang X. Zhang Q. Zhu L. Bian X. (2014). Meta-analysis of interactions between arbuscular mycorrhizal fungi and biotic stressors of plants. Sci. World J. 2014:746506. 10.1155/2014/746506 24558327 PMC3914602

[B107] Yang H. Xu J. Guo Y. Koide R. T. Dai Y. Xu M. (2016). Predicting plant response to arbuscular mycorrhizas: the role of host functional traits. Fungal Ecol. 20, 79–83. 10.1016/j.funeco.2015.12.001

[B108] Yang H. Zhang Q. Dai Y. Liu Q. Tang J. Bian X. (2015). Effects of arbuscular mycorrhizal fungi on plant growth depend on root system: a meta-analysis. Plant Soil 389, 361–374. 10.1007/s11104-014-2370-8

[B109] Zhao R. Guo W. Bi N. Guo J. Wang Zhao J. (2015). Arbuscular mycorrhizal fungi affect the growth, nutrient uptake and water status of maize (*Zea mays* L.) grown in two types of coal mine spoils under drought stress. Appl. Soil Ecol. 88, 41–49. 10.1016/j.apsoil.2014.11.016

